# Chemical and Sensory Evaluation of Silicone and Polylactic Acid-Based Remedial Treatments for Elevated Methoxypyrazine Levels in Wine

**DOI:** 10.3390/molecules21091238

**Published:** 2016-09-16

**Authors:** Andreea Botezatu, Belinda S. Kemp, Gary J. Pickering

**Affiliations:** 1 Cool Climate Oenology and Viticulture Institute, Brock University, St. Catharines, ON L2S 3A1, Canada; bia76@gmx.net (A.B.); bkemp@brocku.ca (B.S.K.); 2 Environmental Sustainability Research Centre, Brock University, St. Catharines, ON L2S 3A1, Canada; 3 Department of Biological Sciences, Brock University, St. Catharines, ON L2S 3A1, Canada; 4 National Wine and Grape Industry Centre, Charles Sturt University, Locked Bag 588, Wagga Wagga, NSW 2678, Australia; 5 Sustainability Research Centre, University of the Sunshine Coast, Sippy Downs, QLD 4556, Australia

**Keywords:** methoxypyrazines, ladybug taint, silicone, polylactic acid

## Abstract

Alkylmethoxypyrazines (MPs) are a class of compounds that can elicit undesirable aroma and flavor characteristics in wine, and resist remediation using traditional wine making approaches. MPs are grape-derived constituents as well as contaminants from *Coccinellidae* beetles present during wine processing; the latter eliciting an off-flavor referred to as ‘ladybug taint’. In this study we investigated the capacity of two plastic polymers—one silicone-based, the other polylactic acid-based—applied with varying surface areas to reduce concentrations of isopropylmethoxypyrazine (IPMP), *sec*-butylmethoxypyrazine (SBMP) and isobutylmethoxypyrazine (IBMP) in a Merlot wine using multi-dimensional gas chromatography coupled with mass spectrometry and headspace solid phase microextraction (SPME-MDGCMS). The impact of treatments on the sensory characteristics of the wine (descriptive analysis) and volatile aroma compounds (VOCs) (SPME-MDGCMS) was also investigated. Results showed substantial reductions for all of the target odorants: up to 38%, 44% and 39% for IPMP, SBMP and IBMP, respectively, for the silicone polymer, and up to 75%, 78% and 77% for IPMP, SBMP and IBMP, respectively, for the polylactic acid polymer. These polymers had no or minimal effect on VOCs at applications of 200 cm^2^/L for silicone or for all polylactic acid treatments. Sensory impacts were less clear, but generally showed minimal effect from the treatments. Taken overall, the data confirm the utility of both polylactic acid and silicone polymers in reducing elevated levels of grape-derived MPs, as well as potentially improving wine contaminated by ladybug taint.

## 1. Introduction

Wine aroma and flavor are influenced by hundreds of volatile compounds from many chemical classes (e.g., esters, aldehydes, alcohols, fatty acids, terpenes) but flavor and aroma balance are impacted when some compounds accumulate at higher than optimal concentrations for a particular wine variety or style. 3-Alkyl-2-methoxypyrazines (MPs) are powerful odorants, commonly found in the natural world. MPs are found in many wine grape varieties such as Cabernet sauvignon, Cabernet franc, Carmeneré, Sauvignon blanc and Chardonnay [1,2], and they can contribute to wine typicity and complexity. However, at higher levels they are responsible for under-ripe, herbaceous and vegetative notes that are considered undesirable attributes in wines. MPs can also be introduced into wines from members of the *Coccinellidae* species, particularly *Harmonia axyridis* and *Coccinella septempunctata* during grape harvest and processing [3,4,5]. Extraction of MPs from these beetles during winemaking procedures can lead to off-flavors known collectively as ladybug taint [3]. The taint in juice and wine is characterized by detrimental aromas and flavors that include “peanuts”, “asparagus”, “green bell pepper”, and “canned green beans” [3]. MPs are very potent, with olfactory perception thresholds ranging between 0.3–16 ng/L, and concentration ranges of 0–60 ng/L reported in commercial wines. Four MPs have been identified as naturally occurring in wines: *iso*-propyl-methoxypyrazine (IPMP), *sec*-butylmethoxypyrazine (SBMP) *iso*-butylmethoxypyrazine (IBMP) and dimethylmethoxypyrazine (DMMP) [2]. IPMP, SBMP and IBMP have all been linked to ladybug taint, with IPMP being the main contributor [4,5,6].

*Harmonia axyridis* populations in Canada, USA and Europe have increased during the last decade, a phenomenon partly attributed to climate change, whereby warmer winters are allowing populations to survive in larger numbers. This has translated into higher numbers of beetle infestation events during harvest in many vineyards in these regions [7]. Additionally, cooler, wetter autumns and years with extreme temperature variations may also be contributing to increased MP levels in grapes and their subsequent wine [8,9].

MPs are relatively stable during alcoholic fermentation and wine ageing, necessitating remediation in cases of high MP must or wine. Several remedial options have been examined in the literature, with varying degrees of success reported. Juice clarification can reduce MP loads by up to 50% [10,11,12,13]. Similarly, thermo-vinification and juice heating can be moderately successful (up to 50% MP reduction) [14], but may lead to undesirable sensorial changes in wines [15]. Selective yeast usage was shown to be ineffective or even detrimental, with one commercial yeast strain increasing MP concentrations [16]. Bentonite fining does not remove MPs, and although fining with charcoal is moderately effective, it lacks the necessary selectivity [11]. Oak chips can partially mask ladybug taint without affecting MP concentrations [11], but are not suitable for many wine styles.

Pickering et al. [17] reported that wines kept in contact with synthetic wine closures had lower levels of MPs compared to control wines. Synthetic closures are typically comprised of a combination of plastic polymers, and the MP reduction observed was putatively attributed to the sorptive properties of these polymers. Similarly, Ryona et al. [18] reported a decrease in MPs in wines produced from juices treated with silicone tubing, likely due to the sorptive properties of silicone. Therefore, sorption onto select, food-grade polymers may represent a general and promising strategy for managing juices and wines with high MP concentrations; however such an approach may also potentially lead to a reduction in beneficial volatile aroma compounds (VOCs). Botezatu and Pickering [19] evaluated the ability of thirteen plastic polymers to reduce MP concentrations in wine, and found in these initial trials that silicone- and polylactic acid-based polymers showed the most promise, with reported reductions of IPMP and IBMP of up to 96% and 100% for silicone, and 53% and 36% for the polylactic acid-based polymer. However, the effects of these treatments on sensory quality and non-target volatile compounds were not reported.

The current study seeks to assess the efficacy of silicone and polylactic acid polymers in remediating MP levels in red wine, with a focus on determining optimum surface areas. Importantly, we also measure the impact of these treatments on VOCs and the sensory properties of treated wines. The findings will establish the selectivity of these polymers, and assist in defining optimum conditions for their use in remediating wines with elevated MP concentrations.

## 2. Results and Discussion

The main aims of this study were to evaluate the ability of silicone and polylactic acid polymers to reduce the concentrations of MPs in red wine, determine how this varies with polymer surface area, and elucidate the effects on other volatile aroma compounds and sensory quality. Sensory analysis was performed on the subset of treated wines where a reduction in MP concentration was observed. The treated wines were labeled silicone 50, silicone 200, silicone 600, polylactic acid (PLA) 50, polylactic acid 200 and polylactic acid 600 based on the surface areas tested (50, 200 or 600 cm^2^/L). A control wine without MP addition (“control”) and a control wine spiked with 20 ng/L each of IPMP, SBMP and IBMP (“control spiked”) were included in the trial. 

### 2.1. Chemical Analyses Results

#### 2.1.1. Methoxypyrazines

**Figure 1:**
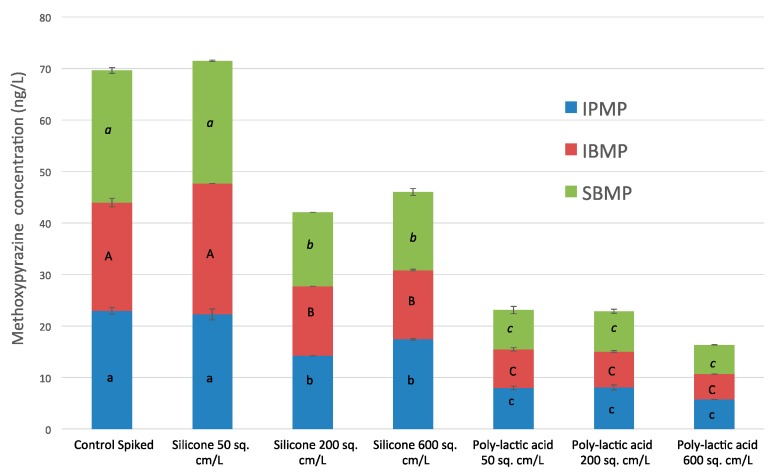
Change in *iso*-propylmethoxypyrazine (IPMP), *iso*-butylmethoxypyrazine (IBMP), and *sec*-butylmethoxypyrazine (SBMP) concentrations after application of silicone or polylactic acid polymers of varying surface areas. Data represent mean values ± standard deviations. Wines with different letters have significantly different concentrations (Tukey’s HSD_0.05_).

As shown in Figure 1, the greatest decrease across all three MPs for the silicone polymer was found using a 200 cm^2^/L surface (IPMP 38%, SBMP 44% and IBMP 39%). For PLA, the most effective surface area was 600 cm^2^/L with reductions of 75%, 78% and 77% for IPMP, SBMP, and IBMP, respectively. However, in the case of PLA, sizeable reductions were also seen with the smallest surface area tested (50 cm^2^/L), with 65.3% less IPMP, 70.3% less SBMP and 66.8% less IBMP detected in treated wine compared with the control spiked wines. Significant differences (ANOVA, *p*(*F*) ≤ 0.05) were found between most of the treatments and control spiked, as indicated in Figure 1. The effectiveness of silicone in removing MPs in wine shown here agrees with Botezatu and Pickering [19], while it is about half as effective as the decrease reported by Ryona et al. [18] for IPMP in grape juice. Botezatu and Pickering [19,20] have previously shown some reduction in MPs in wine from treatment with PLA, although the decrease in SBMP concentrations shown here is significantly larger than previous reported. Overall, the results suggest that the surface area of the polymer plays a role in the efficiency of MP removal, however, with consideration of the findings of Botezatu and Pickering [19], it appears less important than the total contact time between the polymer and wine, particularly for PLA.

#### 2.1.2. Other Volatile Aroma Compounds

**Figure 2:**
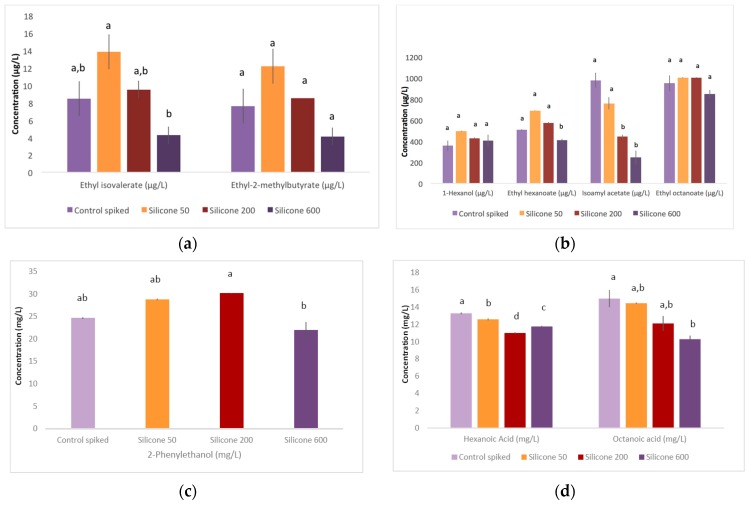
Concentrations of (**a**) ethyl isovalerate, ethyl 2 methylbutyrate; (**b**) ethyl butyrate, hexanol, ethyl hexanoate, isoamyl acetate, ethyl octanoate; (**c**) phenylethanol and (**d**) hexanoic acid and octanoic acid in untreated (‘control spiked’) wine and wine treated with silicone of varying surface areas (50 cm^2^/L, 200 cm^2^/L and 600 cm^2^/L). Data represent mean values ± standard deviations. Wines with different letters have significantly different concentrations for each compound (Tukey’s HSD_0.05_).

**Figure 3:**
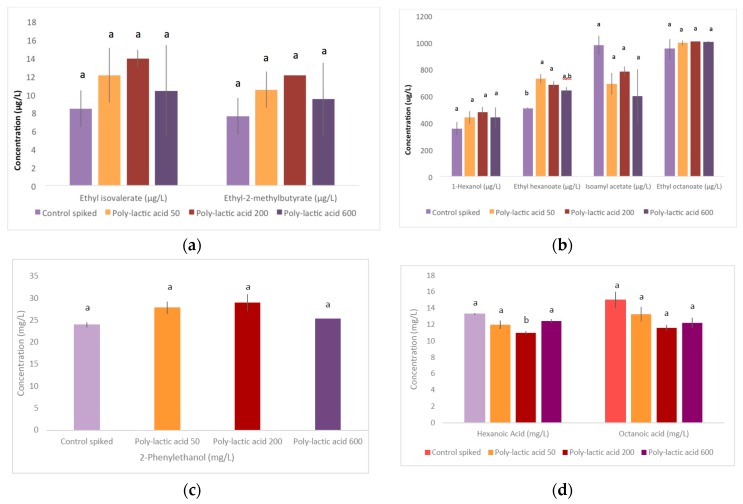
Concentrations of (**a**) ethyl isovalerate, ethyl 2-methylbutyrate; (**b**) ethyl butyrate, hexanol, ethyl hexanoate, isoamyl acetate, ethyl octanoate; (**c**) phenylethanol and (**d**) hexanoic acid and octanoic acid in untreated (‘control spiked’) wine and wine treated with polylactic acid of varying surface areas (50 cm^2^/L, 200 cm^2^/L and 600 cm^2^/L). Data represent mean values ± standard deviations. Wines with different letters have significantly different concentrations for each compound (Tukey’s HSD_0.05_).

Concentrations of volatile compounds measured in silicone and polylactic acid treated and control spiked wines are presented in Figure 2 and Figure 3, respectively. Overall, the treatments had relatively little impact on VOCs, with the exception of silicone at 600 cm^2^/L, where a trend of lower concentrations was observed, reaching significance in the case of ethyl hexanoate, isoamyl acetate, hexanoic acid and octanoic acid. For PLA, ethyl hexanoate increased significantly for the 50 cm^2^/L and 200 cm^2^/L treatments (Figure 3b), and trended in the same direction for ethyl isovalerate and ethyl-2-methylbutyrate (Figure 3a). This may be attributable to the wine’s ethanol and acid matrix that may have contributed to the release of esters [21]. Several reactions have been proposed in the degradation process of PLA polymers, including hydrolysis, depolymerisation, oxidative degradation, and both inter- and intra-molecular transesterification to monomeric and oligomeric esters [22,23].

Several esters increased after silicone treatment in the 50 cm^2^/L application, but the trend was not retained at higher surface areas. The negligible change observed for most other volatiles in the silicone treatment was unexpected, as we expected some scalping would occur.

The scalping of aroma compounds by non-polar polymers used in highly non-polar silicone-based packaging is primarily governed by log *p* values (typically determined at 20 °C) [24]. It is possible that an absorptive phenomenon under kinetic control was the reason for the limited impact of silicone on VOCs (e.g., rigid pyrazines may have migrated faster than the less rigid compounds). Silicone (PDMS) coatings are used to adsorb VOCs in some analytical methods (e.g., Stir Bar Sorptive Extraction and Solid Phase Micro-Extraction), but these applications typically employ higher temperatures and shorter contact time with the silicone than in our study. It is also possible that other aroma compounds not measured in our study had a preferential affinity towards silicone. Finally, the reason for the relative selectivity of silicone for isoamyl acetate compared with its structural isomer ethyl isovalerate remains unclear.

#### 2.1.3. Color and Lactic Acid Analyses

The results for the color analyses are presented in Table 1. Significant although small differences between treatments were found for some of the color parameters (ANOVA; *F* = 15.5 *p* < 0.05), however overall, treatment with polymers had minimal effect. Lactic acid concentrations in PLA treated and control wines (g/L ± SD) were also determined as follows: control spiked, 0.52 ± 0.2; 50 cm^2^/L, 0.53 ± 0.2; 200 cm^2^/L, 0.54 ± 0.1; 600 cm^2^/L, 0.43 ± 0.2. The decrease in lactic acid concentration in the PLA 600 treatment may be due to absorption of lactic acid by the polymer.

### 2.2. Sensory Analysis

Unexpectedly, no significant differences were found between treatments for any of the individual sensory descriptors, although the silicone treatments showed a trend of lower intensity ratings for “green beans”, “green pepper” and “leather/barnyard” aroma (data not shown). The intensity score for the ‘ladybug taint overall’ aroma construct was modestly but significantly lower for silicone 200 compared to the control spiked treatment (*F* = 2.02, *p* = 0.04).

The failure of the analysis of variance to discriminate between treatments was surprising, particularly given the sizeable variation in MP concentrations in several of the wines. The panel consisted of eight assessors, which may have led to the analysis being statistically underpowered and enhancing the likelihood of Type II error. Further, sensory work with methoxypyrazines is often challenging, as adaptation occurs quickly and masking effects from other volatiles have been noted [11]. Nonetheless, seven panel training sessions were conducted in total using established techniques from the descriptive analysis literature, and the panel’s performance overall was considered satisfactory. Finally, the panel does not appear to be insensitive to MPs, as the intensity scores for individual MP-associated attributes were higher in Control Spiked wines than in Control wines, indicating it could differentiate high-MP wines from no-MP wines. Specifically, the average intensity scores (Control, Control Spiked) were: “green pepper”: 5.2, 6.6; “green beans”: 5.7, 6.8; “leather/earthy”: 3.4, 4.4 and “dusty/dirty”: 2.9, 3.7.

## 3. Experimental Section

### 3.1. Wines

A commercial Merlot wine with MP levels below the limit of quantification (<5 ng/L, IBMP; <3 ng/L, SBMP and IPMP; Botezatu et al. [4]) was donated by a Niagara winery for the experiments. Basic physio-chemical parameters of the wine were: ethanol, 12.6% *v*/*v* (GC-FID); pH, 3.58 (VWR 89231-588 benchtop pH meter with a high-flow electrode and ATC temperature correction); titratable acidity, 5.2 g/L (manual titration of a 10 mL aliquot to a pH 8.2 endpoint with 0.1 N NaOH); acetic acid, 0.68 g/L (enzyme kit K-ACET, Megazyme, Bray, Ireland); and total reducing sugars (glucose + fructose), 1.3 g/L (enzyme kit K-FRUGL, Megazyme). The wine was spiked with 20 ng/L of IPMP, SBMP and IBMP. This concentration can be found in wines affected by ladybug taint as well as in wines produced from under ripe grapes, particularly for IBMP and IPMP [25]. It is also sufficient to cause a negative change in the aroma/flavor profile of the wines, yet potentially low enough for remedial treatments to remove MP-impacts to below sensory detection thresholds.

### 3.2. Treatments

We investigated the performance of two FDA approved plastic polymers, silicone (McMaster-Carr, Cleveland, OH, USA, 40 Duro) and a polylactic acid-based polymer (Brantwood Plastics, St. Louis, MO, USA, 50 microns), each at three surface areas (50 cm^2^/L, 200 cm^2^/L and 600 cm^2^/L). The corresponding volumes of these treatments (50 cm^2^/L, 200 cm^2^/L and 600 cm^2^/L) for silicone and PLA respectively, were: 0.4 cm^3^, 0.25 cm^3^; 1.6 cm^3^, 1 cm^3^; and 4.8 cm^3^, 3 cm^3^. PLA is produced by either bacterial fermentation of carbohydrates or by chemical synthesis, and represents a biodegradable, compostable, thermoplastic polymer produced from renewable sources [21]. One polymer (silicone or PLA) and one surface area represent one treatment. 11 L glass carboys were spiked with 20 ng/L of each of the MPs, and a treatment applied by submerging the polymer in the wine for 6 h. Treated, untreated (‘Control’) and MP-spiked + untreated (‘Control spiked’) wines were bottled immediately after the completion of the trials in commercial glass bottles using natural cork and used for sensory and chemical analyses.

### 3.3. Analysis Methods

#### 3.3.1. Methoxypyrazine Analysis

Commercial IBMP (97%), SBMP (99%) and IPMP (97%) and deuterated IBMP (99%) were purchased from Sigma Aldrich (Sigma Aldrich Inc., St. Louis, MO, USA) and MilliQ (EMD Millipore, Billerica, MA, USA) water was used for dilutions. Sodium hydroxide (NaOH 99.8%) was purchased from Sigma Aldrich while NaCl (99%) was purchased from Caledon Laboratories Ltd (Caledon, ON, Canada). The analytical method used for MPs was that of Botezatu et al. [4]. The method yielded good linearity of standard curves for all three MPs (*r*^2^ > 0.99) and satisfactory repeatability and recovery values. The limit of detection was 1–3 ng/L for all MPs, while the limit of quantification was <5 ng/L for IBMP, and <3 ng/L for SBMP and IPMP. MP analyses were performed using an Agilent 7890A Gas-Chromatograph (GC) coupled with an Agilent MS 5975 Mass Spectrophotometer (MS) (Agilent Inc., Santa Clara, CA, USA). The GC was equipped with a Dean Switch (Agilent Inc.) that allowed the flow between the two columns of the multi-dimensional system. The first column was a HP5MS column (30 m, 0.25 mm I.d., 0.25 μm film thickness) (J&W Scientific, Folsom, CA, USA) and the second column was a DBWAX column (30 m, 0.25 mm I.d., 0.25 μm film thickness) (J&W Scientific). The column temperature was held at 70 °C then ramped to 100 °C at a rate of 6 °C/min, further ramped to 130 °C at a rate of 2 °C/min and to 240 °C at a rate of 35 °C/min where it was held for 8 min. The front inlet was set to splitless mode and held at 250 °C under 23.2 psi pressure. The purge flow was turned on after 3 min with a flow to the split vent of 50 mL/min. The dean switch was programmed for two heart cuts, the first between min 10.1 and min 13.5, the second between min 14.1 and min 14.88. The MSD interface was held at 240 °C and the temperature of the ion source was 200 °C. The carrier gas was helium, with an initial flow of 0.8 L/min. Identification was performed using Selective Ion Monitoring (SIM). Mass channels were *m/z* = 137 (quantifying) and 152, for IPMP, 138 (quantifying) and 124 for SBMP, 124 (quantifying) and 151 for IBMP. Mass channels for the internal standard d_3_ IBMP were *m/z* = 127(quantifying) and 154. Quantitation of the compounds was performed by interpolating of the peak area for each compound of interest with the internal standard peak area, based on calibration curves established using the commercial pure compounds and the commercial Merlot wine used throughout the experiments. Quantification of each compound was performed using Chemstation software (MSD E.02.00493, Agilent Technologies). Analyses were performed in duplicate.

#### 3.3.2. Analysis of Volatile Aroma Compounds (VOCs)

A set of representative VOCs from chemical classes important to wine flavour were selected for analysis, and are presented in Table 2, together with their previously reported aroma descriptors, aroma detection thresholds, purity, CAS number and supplier details. The method used for the preparation of VOC standards followed Kemp [26]. Concentrations of standards were prepared according to their expected concentrations using ranges reported in published wine literature. Milli-Q water was obtained from Biocel MilliQ (EMD Millipore, Billerica, MA, USA) and filtered through 0.22 μM filter (Millipore). All stock standard solutions (Standard A) were prepared using ethanol Chromasolv^®^ HPLC standard (Sigma-Aldrich, Oakville, ON, Canada). From Standard A, a composite standard solution was made (Standard C) which was used to prepare a working standard. Each reference compound was identified by its EI spectrum according to Chemstation/Wiley spectral databases (NIST 08). These compounds were also confirmed using qualifiers and quantifiers. The deuterated internal standards were analyzed by EI-MS and matched to the GC-MS EI spectrum.

#### 3.3.3. Preparation of Standards of Volatile Aroma Compounds (VOCs)

To a 20 mL round-bottomed amber glass vials (MicroLiter Analytical Supplies Inc., Millville, NJ, USA), 3 g of reagent grade NaCl (Bioshop, Burlington, ON, Canada) and a magnetic stir bar were added, then 8.06 mL of Milli-Q water followed by the ethanol/water matrix, along with composite aroma standards (Standard C) according to the calibration range in Table 3. 40 μL of ethyl hexanoate-*d*_11_ Solution C was added and the vial was immediately capped with a magnetic screw/thread headspace cap (PTFE/silicone; MicroLiter).

#### 3.3.4. Preparation of Standards of Volatile Fatty Acids

The method of Tomasino [31] for the preparation of volatile fatty acid standards was used, which generally followed that described above for VOCs. Standard A and Standard C composite standards were made up fresh on the day of analysis and the Milli-Q water and matrix were acidified to pH 3.6 with 1 M HCl. Vials were closed with Sun-sri magnetic screw/thread headspace cap PTFE/silicone closures and octanal-*d*_16_ was used as the internal standard.

#### 3.3.5. Sample Preparation

To a 20-mL amber round-bottomed glass vial, 3 g NaCl and a stir bar were added, followed by 8.06 mL of Milli-Q and 0.90 mL of wine for a 10-fold dilution. Finally, 40 μL of the deuterated internal standard ethyl hexanoate-d_11_ Standard C was added and the vial was closed with a magnetic screw cap immediately. Samples were then incubated at 40 °C for 1 min at 600 rpm. For fatty acids, additional dilution was required; wine samples were diluted 50 fold with the acidified matrix. To prepare the sample, 0.90 mL of the diluted wine was added to a 20 mL amber round-bottomed glass vial containing a magnetic stir bar together with 3 g NaCl and 8.06 mL of acidified Milli-Q water. 40 μL of octanal-*d*_16_ Standard C was added immediately and capped with a magnetic screw/thread headspace cap PTFE/silicone.

#### 3.3.6. Head Space-Solid Phase Micro-Extraction-Gas Chromatography (HS-SPME-GC-MS) Parameters for VOCs

A 2 cm divinylbenzene/carboxen/polydimethylsiloxane (DVB/CAR/PDMS) (Supelco Inc., Bellefonte, PA, USA), 23 gauge SPME fiber was used for sampling. Samples were incubated at 40 °C with a conditioned stir bar before exposing the fiber for 30 min at 40 °C at 600 rpm. The samples were analysed using an Agilent (Mississauga, ON, Canada) 7890A gas chromatograph coupled to a 5975C mass selective detector (MSD) equipped with a Gerstal MPS2 XL autosampler (Linthicum Heights, MD, USA). The GC was equipped with a Deans Switch and two columns: a HP-5MS 5% phenyl methyl siloxane column (30 m, 0.25 mm i.d., 0.25 μm film thickness) coupled with a secondary DB-Wax capillary column (30 m, 0.25 mm i.d., 0.25 μm film thickness) (J&W Scientific). The liner was a SPME inlet liner (0.7 mm i.d.; Supelco). Helium was used as the carrier gas with and a flow rate of 0.5 mL/min in the first column, and 1.5 mL/min in the second column. Oven temperature programming began at 35 °C for 3 min, and then increased 3 °C/min up to 105 °C where it was held for 10 min. Temperature was then increased by 2 °C/min up to 140 °C, before holding for 10 min. Temperature went through one more ramp up of 4 °C/min up to 250 °C, before holding for a final 10 min. The run time for this method was 101 min. The MSD interface was held at 250 °C. The inlet temperature was 250 °C and the SPME fiber was desorbed in splitless mode. The solvent delay was 5 min. The fiber was prebaked for 10 min and post baked for 20 min. Samples were warmed at 40 °C and stirred at 600 rpm for 1 min before being exposed to the fiber for 30 min at 40 °C with stirring at 600 rpm, followed by desorption in the inlet for 10 min. Electron ionisation source was used, with a source temperature of 230 °C and electron energy of −70 eV. The samples were measured using synchronous scan and selected ion monitoring (SIM mode). The scan parameters ran from 35 *m/z* to 400 *m/z*, and both scan and SIM acquisitions were performed with an EMV Gain Factor of 7. All analyses were carried out in duplicate.

#### 3.3.7. Head Space Solid Phase Micro-extraction-Gas-Chromatography-Mass Spectrometry (HS-SPME-GC-MS) Parameters for Volatile Fatty Acids

The parameters for volatile fatty acids are described above with the following changes. The oven program was set to 50 °C for 3 min, and then an increase of 10 °C/min to 240 °C followed by 30 °C/min to 250 °C and held for 5 min. The data was collected in scan mode only. The fiber was prebaked for 3 min and post baked for 15 min. The run time was 27 min. All analytes, together with their retention times (RT), qualifying and quantifying ions, calibrations ranges, standard curves, % recovery, limits of detection (LODs) and limits of quantitation (LOQs) are presented in Table 3.

#### 3.3.8. Data Processing

The analytical data software used was Chemstation (MSD E.02.00.493) by Agilent Technologies. The quantifying ions (Table 3) were extracted, and the ratio of the standard over the internal standard was plotted against each analyte concentration to fit a quadratic equation where the intercept was set to zero. Triplicate spiked samples were prepared and analyzed after every 16 wine samples to calculate the recovery.

#### 3.3.9. Other Methods

Wine colour was assessed using the spectrophotometric procedures of Iland et al. [32] using an Agilent Cary 60 UV-VIS spectrophotometer (Agilent Technologies). Lactic acid concentrations in PLA-treated and control wines were also determined, using an enzyme kit (Megazyme).

#### 3.3.10. Sensory Analysis

Wines from treatments that displayed a reduction in MP concentrations were included, along with ‘control’ and ‘control spiked’ wines, in a descriptive analysis. The analysis was performed after seven training sessions by a panel of 8 judges (ages 20–45, 2 males, and 6 females) recruited from students and staff at the Cool Climate Oenology and Viticulture Institute (CCOVI). Research Ethics clearance was granted by the Research Ethics Office (File No. 13-028) at Brock University. Wines (30 mL) were presented in clear ISO glasses at room temperature, covered with Petri dish lids and labeled A-E for the training sessions. Bottles were opened one hour prior to evaluation and checked for possible faults (i.e., oxidation or cork taint). In the first two training sessions the judges were presented with all the wines and were asked to generate lists of descriptors for both aroma and taste modalities. From the descriptors generated an abbreviated list was created with the approval of all judges by eliminating redundant, repetitive or overlapping terms. The final descriptor list consisted of: red fruit, dark fruit, dried fruit, green pepper, green beans, spice, herbal, olives, dusty/dirty, leather/earthy/barnyard, candy/medicinal and peanuts for aroma evaluation and red fruit, dark fruit, green/vegetal, spice, briny, leather/earthy, grassy green and candy for flavor evaluation. For basic tastes and in-mouth sensations; sourness, bitterness, heat and astringency were rated. Sweetness was not evaluated because judges did not distinguish sweetness differences between wines during the initial two training sessions.

Following the initial two training sessions a further five were undertaken. Judges were presented with aroma standards for all aroma descriptors. Most of the standards were prepared using a Wine Awakenings^®^ Kit (Picksen International, Port Colborne, ON, Canada) by mixing the Merlot base wine with various aroma standards, as presented in Table 4. The judges were trained to rate all descriptors in a uniform manner, using 15 cm scales anchored with the terms *Absent* and *Very High*, on paper ballots. After five training sessions the ability of the judges to evaluate the wines as a panel was considered satisfactory. The wines were formally evaluated in the Sensory Laboratory at CCOVI, under controlled room temperature and humidity conditions. Data collection was performed using a computerized system equipped with Compusense5^®^ (Compusense Inc., Guelph, ON, Canada) software. Each judge was seated in an individual booth and recorded all evaluations using the Compusense5^®^ software system. Judges were presented with the seven wines included in the trial using a complete randomized block design, and each wine was labeled with a 3 digit identifier code. The wines were presented for each data collection session in two flights of four and three wines respectively. The judges were instructed to evaluate the wines in the order presented. There were two min forced breaks between each wine and a 15-min break between flights. Judges were instructed to cleanse their palates as needed with distilled water and plain unsalted crackers (President’s Choice^®^ Soda Crackers). All wines were evaluated in triplicate, and only one evaluation session was performed per day. 

#### 3.3.11. Statistical Analysis

All statistical analyses were performed using XLSTAT software version 2015.01 (Addinsoft, Paris, France). For the sensory data, Analysis of Variance (ANOVA) was performed (*p* = 0.05), with intensity ratings as the dependent variable, and wine, judge, replicate and 2-way interactions as independent variables. Two composite variables were also created and analyzed: Ladybug taint (LBT) overall and Fruit overall. These new constructs were created by averaging intensity ratings for several descriptors for each construct. In the case of LBT overall aroma the descriptors amalgamated were Green pepper, Green beans, Olives, Leather/Earthy and Dusty/Dirty. For LBT overall flavour they were Green/Vegetal and Grassy green. For Fruit overall aroma the descriptors amalgamated were Red fruit, Dark fruit and Dried fruit, and for Fruit overall flavour they were Red fruit and Dark fruit. Principal Component Analysis, without rotation, was also performed on all the sensory data. Differences between wines for VOC concentrations were also assessed using ANOVA, with Tukey’s HSD_0.05_ as the means separation test. 

## 4. Conclusions

A reduction in the concentrations of all three MPs of between 38%–44% for the silicone polymer (200 cm^2^/L) and 75%–78% for the polylactic polymer (600 cm^2^/L) was observed. These changes represent a significant decrease in the MP load of treated wines, and compare very favorably with the few other remedial treatments suggested in the literature. Importantly, very little change in most non-target volatile aroma compounds and color parameters was found, partially addressing potential concerns regarding the selectivity of these polymers. Sensory results were less clear, often not showing the expected reduction in intensity of descriptors associated with MPs, possibly due to differential masking phenomena that have previously been reported with MP remediation [11,16], and/or the analysis being underpowered. Importantly, the sensory data demonstrated that the polymers did not contribute negatively to the aroma or flavor profiles of treated wines. Consideration of the combined results suggests that polylactic acid at 600 cm^2^/L or silicone at 200 cm^2^/L are the preferred treatments for red wine under the conditions assessed here. 

Further research is encouraged to better understand the effect these polymers may have on juice, white wine, and other wine styles, as well as the potential for compounds to leach into treated product. Practical consideration and evaluation of how these polymers may best be incorporated into commercial juice and wine processing are needed. For instance, poly-lactic acid can be produced in a variety of forms with different physical properties, conferring flexibility in how it might be used. Potentially, it could be integrated into existing filtration systems, manufactured as solid tank inserts, or added as pellets directly to the product and later removed when the desired reduction in MP content has been achieved.

## Figures and Tables

**Table 1:** Color parameters for untreated (‘control spiked’) wine and wine treated with poly-lactic acid or silicone of varying surface areas (50 cm^2^/L, 200 cm^2^/L and 600 cm^2^/L) determined through spectrophotometric analyses. Data represent mean values * ± standard deviations.

Treatment	SO_2_ Resistant Pigments (A.U.)	Total Red Pigments (A.U.)	Wine Color Density (A.U.)	Wine Hue (A.U.)	Red Pigment Coloration (A.U.)	Total Phenolics (A.U.)
Control spiked	2.89 ^a,b^	12.37	6.16 ^a,b^	0.83	0.27	49.53 ^a,b^
Polylactic acid 50	2.92 ^b^	11.17	6.07 ^a^	0.83	0.30	50.73 ^b^
Polylactic acid 200	2.89 ^a,b^	11.35	6.14 ^a,b^	0.85	0.30	50.85 ^b^
Polylactic acid 600	2.81 ^a^	10.97	6.02 ^a^	0.86	0.30	48.63 ^a^
Silicone 50	2.87 ^a,b^	13.03	6.11 ^a,b^	0.85	0.27	50.17 ^a,b^
Silicone 200	3.08 ^c^	10.23	6.39 ^c^	0.87	0.34	50.77 ^b^
Silicone 600	2.95 ^b^	11.00	6.28 ^b,c^	0.86	0.31	50.30 ^a,b^

* Means with different letters are significantly different (ANOVA followed by Tukey’s HSD_0.05_).

**Table 2:** Aroma descriptors, odor detection thresholds, purity, CAS numbers and suppliers of the volatile aroma compounds analysed.

Aroma Compound	Aroma Descriptors	Odor detection Threshold (µg/L)	Purity (%)	CAS No.	Chemical Supplier
d_11_ Ethyl hexanoate ISTD	N/A	N/A	98.7	2159-19-5	CDN Isotopes, Pointe-Claire, Quebec, Canada.
Octanal-*d*_16_ ISTD	N/A	N/A	98	1219794-66-7	CDN Isotopes, Pointe-Claire, Quebec, Canada.
Ethyl octanoate	Fruity, apricot, pineapple	580 ^a^	>99	106-32-1	Sigma Aldrich
Ethyl hexanoate	Apple, blackberry	62 ᵈ	99	123-66-0	Sigma Aldrich
Ethyl butanoate	Acid fruit, candy, strawberry	20 ᵇ	99	105-54-4	Sigma Aldrich
Ethyl isovalerate	Mint, fruit	3 ᶜ	98	108-64-5	Sigma Aldrich
Ethyl-2-methylbutyrate	Sweet fruit	18 ᶜ	99	7452-79-1	Sigma Aldrich
Isoamyl acetate	Banana	30 ᵇ	97	123-92-2	SAFC, St. Louis, MO, USA
2-Phenylethanol	Roses	14,000 ᶜ	99	60-12-8	Sigma Aldrich
1-Hexanol	Herbal, green, grass	8000 ᵇ	99.5	111-27-3	Sigma Aldrich
Hexanoic acid	Cheese, sweaty	420 ᶜ	99.5	142-62-1	Sigma Aldrich
Octanoic acid	Rancid, harsh	500 ᶜ	99.5	124-07-2	Sigma Aldrich

ᵃ Etiévant [27]. Odor thresholds determined in wine, ᵇ Guth et al. [28]. Odor thresholds determined in 10% ethanol/water solution, ᶜ Ferreira et al. [29]. Odor thresholds determined in 10% ethanol/water solution with 7g/L glycerol at pH 3.2, ᵈ San Juan et al. [30]. Odor threshold determined in 10% ethanol/water solution at pH 3.2.

**Table 3:** Volatile aroma compounds, retention times, target and confirming ions, calibration ranges, standard curves, % recovery, % coefficient of variation (CV), limits of detection (LODs) and limits of quantitation (LOQs). LODs were calculated as the mean of the blank plus 3* standard deviation for each analyte in a blank sample. LOQs were calculated as the mean of the blank plus 10* standard deviation for each analyte in a blank sample.

Compound	Retention Time (min)	Target Ion (*m*/*z*)	Confirming Ions (*m*/*z*)	Calibration Range (µg/L)	Standard Curve (R²)	% Recovery	% CV	LOD (µg/L)	LOQ (µg/L)
Ethyl butyrate	15.5	88	101, 60	0.148–62.20	0.9956	74	4	0.670	0.148
Ethyl hexanoate	26.3	88	115, 60	0.417–142.10	0.9979	98	4	0.320	0.417
Ethyl isovalerate	18.2	88	85, 130	0.055–12.70	0.9918	78	2	0.035	0.055
Ethyl octanoate	42.5	88	101, 129	0.536–100.94	0.9935	84	5	0.376	0.536
Ethyl-2-methylbutyrate	18.0	57	102, 130	0.153–5.91	0.9825	88	2	0.078	0.153
Hexanol	22.0	56	55, 84	1.249–433.56	0.9935	82	8	0.660	1.249
Isoamyl acetate	19.5	87	43, 73	1.402–352.20	0.9935	77	2	0.857	1.402
Phenylethanol	50.0	91	88, 122	36.297–5716.10	0.9868	119	13	23.323	36.297
Hexanoic acid	15.8	60	73, 87	2.044–365.87	0.9933	81	6	1.425	2.044
Octanoic acid	18.2	60	73, 101	0.401–365.87	0.9968	97	3	0.319	0.401

**Table 4:** Recipes for aroma standards used for panel training. Recipes were prepared using 500 mL of Merlot base wine and aroma standards from the Wine Awakenings^®^ Kit or other commercial products, as indicated.

Standard	Descriptor	Recipe
1	Red fruit	Wine + 3 drops “cherry” + 2 drops “strawberry”
2	Dark fruit	Wine + 2 drops “fig” + 2 drops “dark cherry” + 1 drop “linden” + 4 drops ”ripe blackberry”
3	Dried fruit	Wine + 3 drops “fig” + 2 drops “prune”
4	Green pepper	Wine + 2 drops “green pepper”
5	Green beans	Wine + 4 tablespoons of green bean brine from a can of President’s Choice^®^ Green Beans
6	Spice	Wine + 2 drops “black pepper” + 2 drops “clove” + 5 drops “baking spice” + drops “anise”
7	Herbal	Wine + 1 drop “eucalyptus” + 1 drops “cedar” + 1 drop “green/herbaceous”
8	Olives	Wine + 4 tablespoons brine form a can of President’s Choice^®^ Green Olives
9	Dusty/Dirty	Wine + 1 drop “mineral/wet rock”
10	Leather/earthy	Wine + 2 drops “ leather” + 1 drop “truffles” + 1 drop “mushroom”
11	Candy/medicinal	Wine + 8 Jolly Rancher candy + 5 Jujubes, blended
12	Peanuts	Wine + 3 tablespoons raw peanuts, blended
13	Grassy/green	Wine + 2 drops “green/herbaceous” + 1 drop “unripe”
14	Brine	Wine + 2 drops “olives” + 1 teaspoon President’s Choice^®^ Soy Sauce
